# Age-related impairments and influence of visual feedback when learning to stand with unexpected sensorimotor delays

**DOI:** 10.3389/fnagi.2023.1325012

**Published:** 2023-12-15

**Authors:** Brandon G. Rasman, Christian van der Zalm, Patrick A. Forbes

**Affiliations:** ^1^Department of Neuroscience, Erasmus MC, University Medical Center Rotterdam, Rotterdam, Netherlands; ^2^School of Physical Education, Sport and Exercise Sciences, University of Otago, Dunedin, New Zealand; ^3^Donders Institute for Brain, Cognition and Behaviour, Radboud University, Nijmegen, Netherlands; ^4^Department of Biomechanical Engineering, Delft University of Technology, Delft, Netherlands

**Keywords:** sensorimotor learning, aging, sensorimotor delay, standing balance, posture, visual feedback, generalization of learning

## Abstract

**Background:**

While standing upright, the brain must accurately accommodate for delays between sensory feedback and self-generated motor commands. Natural aging may limit adaptation to sensorimotor delays due to age-related decline in sensory acuity, neuromuscular capacity and cognitive function. This study examined balance learning in young and older adults as they stood with robot-induced sensorimotor delays.

**Methods:**

A cohort of community dwelling young (mean = 23.6 years, *N* = 20) and older adults (mean = 70.1 years, *N* = 20) participated in this balance learning study. Participants stood on a robotic balance simulator which was used to artificially impose a 250 ms delay into their control of standing. Young and older adults practiced to balance with the imposed delay either with or without visual feedback (i.e., eyes open or closed), resulting in four training groups. We assessed their balance behavior and performance (i.e., variability in postural sway and ability to maintain upright posture) before, during and after training. We further evaluated whether training benefits gained in one visual condition transferred to the untrained condition.

**Results:**

All participants, regardless of age or visual training condition, improved their balance performance through training to stand with the imposed delay. Compared to young adults, however, older adults had larger postural oscillations at all stages of the experiments, exhibited less relative learning to balance with the delay and had slower rates of balance improvement. Visual feedback was not required to learn to stand with the imposed delay, but it had a modest effect on the amount of time participants could remain upright. For all groups, balance improvements gained from training in one visual condition transferred to the untrained visual condition.

**Conclusion:**

Our study reveals that while advanced age partially impairs balance learning, the older nervous system maintains the ability to recalibrate motor control to stand with initially destabilizing sensorimotor delays under differing visual feedback conditions.

## Introduction

To control movement, the brain must accurately associate self-generated motor commands with delayed sensory feedback. Human bipedal activities, like standing, are mechanically unstable (Fitzpatrick et al., 1992; Loram and Lakie, 2002; Morasso and Sanguineti, 2002) and thus failing to accommodate for sensorimotor delays can impede stable balance and ultimately lead to a fall (Kuo, 1995; Bingham et al., 2011; van der Kooij and Peterka, 2011). As humans age, gradual changes in nerve conduction, muscle force generation and neural processing lead to lengthening delays in balance control of ~10–30 ms (Allum et al., 2002; Lin and Woollacott, 2002; Davidson et al., 2011; Wiesmeier et al., 2015). These delays can in turn limit the predicted region of stability for the balance controller (Bingham et al., 2011; van der Kooij and Peterka, 2011; Le Mouel and Brette, 2019). Given that healthy older adults can effectively maintain upright balance, one may infer that the older nervous system can compensate for increasing sensorimotor delays. It remains unknown, however, to what extent the aging nervous system can adapt to prolonged delays that are known to destabilize balance in young participants. Adaptation to longer unexpected sensorimotor delays may be limited by age-related decline in sensory acuity, neuromuscular capacity and cognitive function (Seidler et al., 2010; Wolpe et al., 2016; Lord et al., 2018). In this study, we use a robotic balance simulator to determine whether older age influences the ability to learn to maintain standing balance with added sensorimotor delays and further examine the reliance on visual feedback for balance learning.

In young healthy adults, artificially imposing long delays (i.e., ≥ 200 ms) during standing initially destabilizes upright posture, but through training, participants can regain the ability to balance upright (Rasman et al., 2021). This occurs through learning of the causal relationships between delayed sensory feedback of whole-body movements and self-generated balance motor commands (Rasman et al., 2021). Older age may hinder the ability to adapt balance control with added sensorimotor delays for a variety of reasons. Notably, the quality of balance-relevant sensory information (i.e., acuity of vestibular, visual, somatosensory and auditory signals) degrades with older age (Shaffer and Harrison, 2007; Patel et al., 2009; Lord et al., 2018), and may limit the brain’s ability to re-associate delayed sensory feedback with balancing motor commands. Additionally, the generation of motor commands needed to balance with imposed delays may be compromised due to reduced muscular strength (Larsson et al., 1979; Kallman et al., 1990; Doherty, 2003), slower rates of muscle force production (Larsson et al., 1979; Thelen et al., 1996), longer reflex latencies (Dorfman and Bosley, 1979; Allum et al., 2002) and longer cognitive processing times (Lord and Fitzpatrick, 2001). Furthermore, when cognitive systems must accommodate more challenging balance conditions (Paillard, 2017; Ozdemir et al., 2018), such as standing with increasing delays, older adults may be hindered by age-related decline in cognitive function (Jeka et al., 2006; Lord et al., 2018; Wolpe et al., 2020). Considering these factors, the first aim of the present study was to determine whether learning to balance with long (250 ms) imposed sensorimotor delays differs between young and older healthy adults. As the extent of improvement in performance and rate of learning has been observed to degrade with older age across a variety of motor tasks (Seidler, 2006; Vandevoorde and Orban de Xivry, 2019; Vachon et al., 2020; Wolpe et al., 2020), we hypothesized that older adults would exhibit less overall balance improvement (i.e., reduction of postural oscillations) and learn at slower rates.

Another important consideration for balance learning in young and older adults is the relative importance of different sensory channels. Humans use visual, vestibular, somatosensory and auditory cues in the control of upright balance (Forbes et al., 2018). Theoretically, the brain recalibrates estimates of self-motion when training to balance with imposed delays by utilizing all available sensory cues. The importance of vision for balance control is well demonstrated when individuals stand with their eyes closed, which increases postural sway by 20–70% (Travis, 1945; Day et al., 1993; van der Kooij et al., 2011). Consequently, we questioned whether visual feedback is required for participants to learn to stand with unexpected sensorimotor delays. Given the influence that vision can have on normal standing, we hypothesized that learning to balance with delays in the absence of vision would be reduced (i.e., less overall balance stability and slower learning rates). We further predicted that removing vision would impair balance learning to a greater extent for older adults, since older age has been associated with greater reliance on visual feedback when controlling balance during standing (Lord and Menz, 2000; Bugnariu and Fung, 2007; Jeka et al., 2010) and walking activities (O’Connor and Kuo, 2009; McAndrew et al., 2010; Franz et al., 2015).

**Figure 1:**
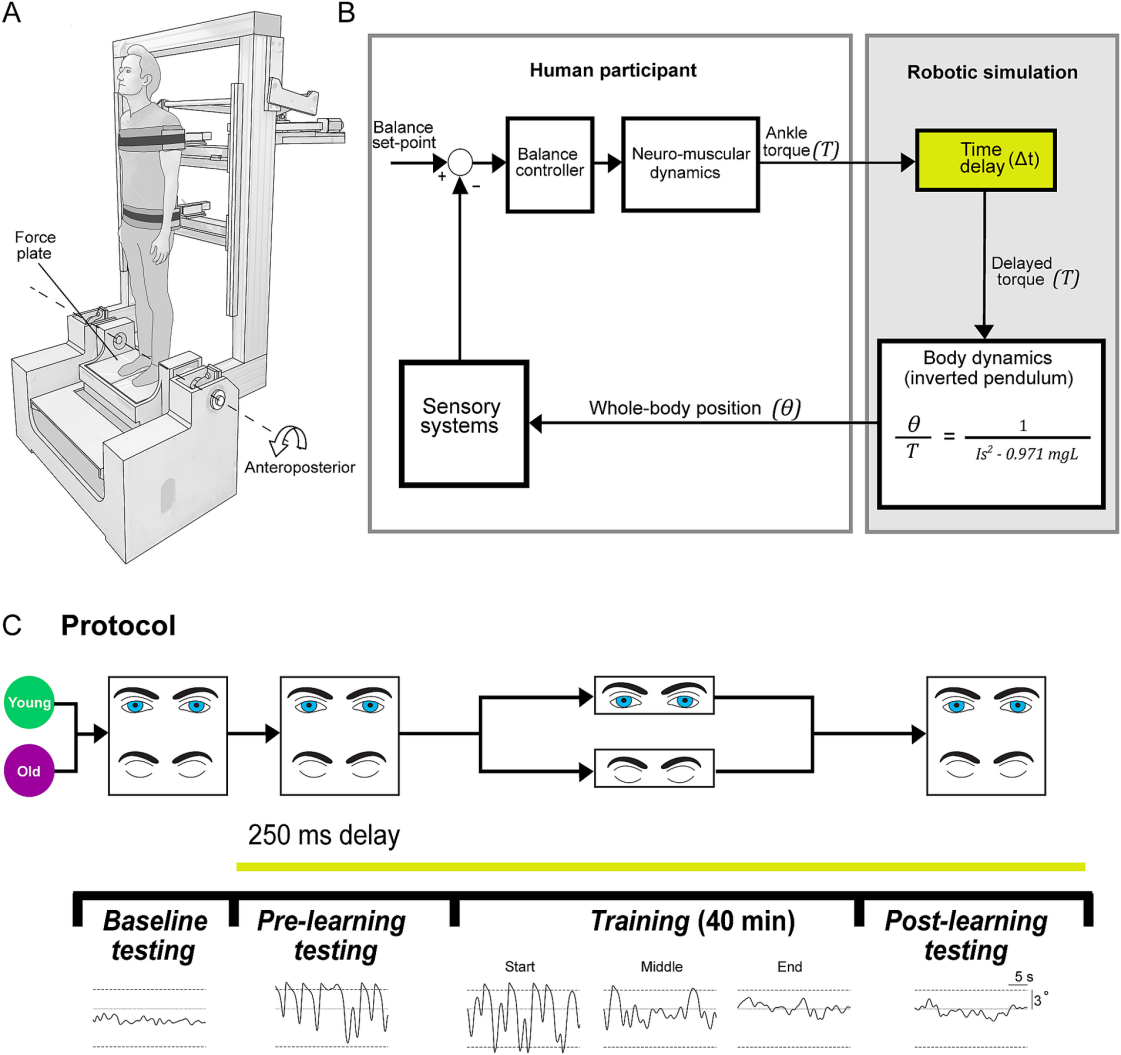
Experimental set-up. **(A)** Experiments were conducted using a robotic balance simulator. Participants stood on a force plate and were secured to the robot frame with torso and hip harnesses. Motion was restricted to the anteroposterior direction and controlled by a linear actuator (see Methods). **(B)** Participants balanced the robotic balance simulator as it operated at baseline (~4 ms delay) or with an imposed 250 ms delay. Delays were imposed by buffering ankle-produced torque signals (T, measured by the force plate) in the robotic simulation computer model such that angular rotation (θ) of the whole-body was dictated by delayed torque. **(C)** Participants (20 young, 20 old) first completed a baseline testing session, where they balanced on the robot in its baseline condition for two 60-s trials (one with vision, one without vision). After baseline testing, all remaining balance trials were performed with the 250 ms delay. Participants then performed a pre-learning session, completing four 30-s trials (two with vision, two without vision). Participants were then separated into either a vision or no vision training group, where they practiced balancing with the 250 ms delay for 40 min (eight 5-min trials). This resulted in four training groups: *young vision, young no vision, old vision, old no vision*. Following training, all participants completed a post-learning session that was identical to the pre-learning session. Traces on the bottom show sample raw data of whole-body angular position in the anteroposterior direction during baseline, pre-learning, training (1st, 20th and 40th minutes; i.e., start, middle and end) and post-learning trials (sample data is from vision trials of a participant from *young vision group*). Dashed lines represent the virtual position limits (6° anterior, 3° posterior).

To test our hypotheses, we conducted a study where young (< 30 years) and older (> 65 years) participants stood on a robotic balance simulator and trained to maintain upright posture with robot-induced delays of 250 ms (Figure 1). Participants first performed normal standing balance trials with the robotic simulator operating in its baseline condition. Young and older adult groups then trained to maintain standing balance with imposed delays either with or without vision (resulting in four training groups). We evaluated balance performance in these groups to determine if and how older age and vision influenced balance learning, and whether these two factors interact. Additionally, we tested whether learning to balance in either visual condition transferred to the opposite condition by examining balance behavior before and after training both with and without vision.

## Materials and methods

### Participants

A total of 40 healthy community-dwelling adults participated in this study. We recruited 20 young (< 30 years) adult participants (11 females and 9 males; mean ± SD for age = 23.6 ± 2.0 years; height = 172.7 ± 9.6 cm; mass = 69.3 ± 17.9 kg; BMI = 23.1 ± 3.0) and 20 older adult (> 65) participants (9 females and 11 males; age = 70.1 ± 3.9 years; height = 175.1 ± 8.0 cm; mass = 79.2 ± 13.2 kg; BMI = 25.7 ± 4.9). We defined older adults as participants ≥65 (though all of our older participants were > 65) based on the definition used by the American Medical Association (Lundebjerg et al., 2017). Participants were recruited from Erasmus University Rotterdam, as well as from surrounding regions of South Holland, The Netherlands. Prior to study enrollment, all participants confirmed they had no known history of neurological and/or balance deficits, no pre-existing neuromuscular injuries, no history of neurological or psychiatric disorders and were not taking acute or chronic psychoactive drugs. Recruitment criteria further required that participants were in overall good physical health (e.g., were physically capable of moderate exercise such as walking for >30 min). All of our participants stated that they engaged in physical activities such as walking or cycling at least three times a week. The experimental protocol was verbally explained before the experiment and written informed consent was obtained. The experiments were approved by the Medical Research Ethics Committee Erasmus MC and conformed to the Declaration of Helsinki.

### Experimental set-up

For all experiments, participants stood on a custom-designed robotic balance simulator programmed to replicate the control of human standing balance in the anteroposterior plane (Figure 1). The mechanical load of the body was simulated as an inverted pendulum (see more details below) using a real-time motion controller (PXI-8880; National Instruments, TX, USA) running at 500 Hz. We programmed the simulation using the anthropometry of each participant, including: mass, center of mass height and ankle height. To measure the height of their center of mass, participants laid supine on a rigid board that was balanced over a round tube positioned transversally under the board. Participants shifted their body longitudinally over the board until the distribution of their mass was balanced. The distance between the ankle joint anteroposterior axis of rotation (Sado et al., 2021) (i.e., anterior edge of medial malleolus) and the tipping point of the board was determined as the height of the center of mass (average: 0.89, SD: 0.04 m). Participants were secured to the robot with harnesses and seat belts at the height of the hips and shoulders (Figure 1A). The harnesses were lined with medium density foam and an additional layer of foam was placed between the seatbelts and the participant. While secured to the robot, participants stood on a force plate (AMTI BP400 × 600; Watertown, MA, USA) which measured ground reaction forces and torques. The force plate was securely mounted on top of an ankle-tilt platform which was kept horizontal (replicating standing on level ground) in all trials. Participants wore noise canceling headphones (WH-1000XM3 Noise Canceling Headphones, Sony, Japan) while listening to audio of garden sounds (water fountain with birds singing) to minimize acoustic cues of motion produced by the motors as well as other extraneous sounds.

Participants were placed inside the control loop of the robot (Figure 1B), such that the position and motion of the robotic system (and thus the participant’s upright body) are driven by participant-generated actions. Participants controlled the real-time balance simulation by modulating ankle plantar/dorsiflexor torque on the force plate. The robotic system consists of a rigid backboard frame, torso and hip harnesses, and an ankle tilt platform, each controlled by separate servo motor-driven linear actuators (Tisserand et al., 2022; Qiao et al., 2023). For these experiments, the torso, hip and ankle-tilt actuators were fixed such that motion only occurred by rotating the backboard (i.e., the whole-body) in the anteroposterior direction. The backboard frame is driven by a 2 kW servo motor (ECMA-J11020S4, Delta, Taiwan; maximum continuous torque: ~6,170 Nm; angular resolution of ~0.0000054°) connected to a 665 mm linear actuator (Y-H1116165P09152A; Rollon, Italy). The robot moves the backboard in response to the applied ankle torque on the force plate that is fed into a computer simulation of standing balance. The robotic system has an ~4 ms delay between a position command and the measured position change of the motors.

The robotic simulator implemented the dynamics of a single-link inverted pendulum to control whole-body motion through a continuous transfer function converted to a discrete-time equivalent for real-time implementation using the zero-order hold method


$$I\overset{¨}{\theta }-0.971\text{mgL}\theta =T$$

(1) $$\frac{\theta }{T}=\frac{1}{Is^{2}-0.971mgL}$$

where θ is the angular position of the body’s center of mass relative to the ankle joint from vertical and is positive for a plantar-flexed ankle angular position, T is the ankle torque applied to the body and is positive for a plantar-flexor torque, m is the participant’s mass, L is the distance from the ankle joint to the body’s center of mass, g is gravitational acceleration (9.81 m/s^2^), and I is mass moment of inertia of the body measured about the ankles ($0.971mL^{2}$). Total participant mass was multiplied by 0.971 to approximate the mass above the ankles.

Angular motion of the body was restricted using software limits to a maximum sway position of 6° anterior and 3° posterior to ensure that participants could generate sufficient torque to balance the system across the range of motion (Luu et al., 2011; Forbes et al., 2016; Rasman et al., 2021). When participants exceeded the software position limits, the program gradually increased the simulated stiffness such that participants could not rotate further in that direction regardless of the torques they produced at the ankle. This was performed by linearly increasing a passive supportive torque to a threshold equivalent to the participant’s body load over a range of 1° beyond the simulated balance limits, providing a passive support of the body at that angle. An additional damping term was implemented to ensure a smooth attenuation of motion when participants exceeded the balance limits. Active torque applied by the participants in the opposite direction enabled them to get out of the limits.

In the present experiments, 250 ms delays were added between the participant-generated ankle torque (i.e., motor command) and the resulting whole-body motion (i.e., sensory feedback). Delays were imposed by buffering participant-generated torque and force recordings such that the signals driving motor position commands (thus whole-body motion) could be delivered based on the ground reaction torques participants generated up to 250 ms in the past. It is worth noting that the internal (i.e., physiological) sensorimotor delays within young adult standing balance control are ~100–160 ms (van der Kooij et al., 1999; Kuo, 2005; Forbes et al., 2018), with older adults having ~10–30 ms longer sensorimotor delays (Allum et al., 2002; Lin and Woollacott, 2002; Davidson et al., 2011; Wiesmeier et al., 2015). Throughout this study, we refer to the delays added through the robotic simulator (baseline (4 ms) – 250 ms), but note that the net sensorimotor delays (i.e., physiological delay + robot-induced delay) for the standing balance task were ~ 350–440 ms. All participants were naïve to the delay protocols and were simply told that “in some trials, the robotic control will be changed, such that your body movement may seem unexpected or abnormal, and standing balance may become more difficult. However, during these conditions you will still be in control of your own standing movements.” In all experiments, participants were instructed to stand upright normally at their preferred standing angle (typically ~1–2 ° anterior). In trials with imposed 250 ms delays, participants had difficulty maintaining a stable upright posture and would often exceed the simulated balancing limits (i.e., 6° anterior or 3° posterior). Participants were instructed to always get out of the limits and continue to attempt to balance upright. After a trial was completed, the robot was returned to a neutral position (0°) at a fixed velocity (0.5 °/s) in preparation for the next trial.

### Familiarization

For all experiments, an introductory balance session was first completed to familiarize the participant with the control of the robot. Instructions were given on the nature of movement control; i.e., similar to standing, torque applied to the support surface (force plate) will control the motion of the upright body (via the backboard frame). Participants were familiarized with the baseline control of the robot in the anteroposterior direction. In a forward leaning position, plantar-flexor torque is required to stabilize the body and increasing the plantar-flexor torque greater than gravitational torque will cause the body to accelerate backward. Similarly, a dorsi-flexor torque is required to remain standing in a backward leaning position and an increase in dorsi-flexor torque will accelerate the body forward. Participants were also instructed to sway back and forth and allow the robot to reach its position limits (6° anterior, 3° posterior), which occurs if the magnitude of the generated ankle torque is not large enough to resist the toppling torque of gravity. Participants performed this familiarization period until they were accustomed to standing on the robot and could maintain upright posture at these baseline conditions with ease. The familiarization session was completed within ~5 min. After becoming familiar with the control of the robot, participants were then asked to stand quietly and maintain an upright posture (normal standing). We explained to participants that this familiarization trial was the baseline condition of the robot, and that while it may be more difficult to balance upright for some experimental trials, they would always be able to control their motion by adjusting how they loaded and pushed their feet against the force plate.

## Experimental protocol

In our experiments, we designed a training protocol (Figure 1C) to determine the effects of older age and presence of visual cues on balance learning. Participants trained to stand in the anteroposterior plane with an imposed delay of 250 ms. We chose a 250 ms delay because when initially imposing this delay, young and older participants exhibit large increases in postural sway variability and cannot maintain standing balance without reaching the virtual limits for more than a few seconds. Consequently, prolonged training is required to adapt their balance control. This knowledge was based on both previous research (Rasman et al., 2021) and pilot testing. Participants were divided into training groups which either practiced to balance with the imposed delay with vision (eyes open with rooms lights on) or without vision (eyes closed and blindfolded with room lights off), providing four participant training groups: *young vision, old vision, young no vision, old no vision*.

All participants first completed two 60-s baseline trials: one with vision and one without vision. Participants then performed a pre-learning session consisting of four 30-s trials in which they balanced with the 250 ms delay: two with vision and two without vision (see Figure 1C). Trial length was limited to 30 s to minimize potential adaptation and learning to the imposed delay during these trials. Participants then performed a 40-min training session, where they practiced to balance with the 250 ms delay over eight 5-min trials. Rest breaks were given every two training trials, where participants stepped out of the robot and sat in a chair for 4–6 min. After completing the training protocol, participants performed a post-learning session that mimicked the pre-learning session. In all pre-learning and post-learning sessions, we randomized whether participants first experienced a vision or no vision trial and then alternated the conditions.

## Data processing

All non-statistical data processing described below was performed using custom-designed routines in Matlab software (2022a version, Mathworks, Natick, MA, USA).

### Measures of balance behavior

For all participants, we extracted three measures of balance behavior and performance: (1) sway velocity variance (from whole-body angular velocity), (2) ankle torque variance (from ankle-produced torques), and (3) longest time within balance limits (from whole-body sway angular position). Sway velocity variance and ankle torque variance were only estimated from data in which whole-body angular position was within the virtual position limits (6° anterior and 3° posterior) as standing with imposed delays ≥200 ms can result in participants crossing the limits (Rasman et al., 2021). When first standing with a 250 ms delay, participants can typically only balance within the limits for short periods (~2–5 s). Therefore, to extract meaningful sway velocity and ankle torque information throughout the entire trial, we extracted data in non-overlapping 2 s windows when there was at least one period of 2 continuous seconds within the simulated balance limits. The extracted data were limited to multiples of 2 s, such that if there was a 5 s segment of continuous balance, only the first two 2 s windows (i.e., first 4 s of the segment) were extracted. On a participant-by-participant basis, we then averaged sway velocity variance and ankle torque variance estimated from these 2 s windows to provide an estimate of sway velocity variance and ankle torque variance for each participant in each experimental condition (Rasman et al., 2021). In the training trials, sway velocity variance and ankle torque variance were estimated from non-overlapping 2 s windows taken across 1 min intervals. These variance estimates were then averaged for every minute of training and further averaged across all participants, providing a minute-by-minute representation of sway velocity and torque variance in the training trials. For the training data, we further normalized sway velocity variance and ankle torque variance on a participant-by-participant basis by dividing by the maximum value across the 40 min of training. This was done for visual illustration purposes as it aided in the comparison of training data across the four groups. As an additional measure of balance behavior, we computed the longest time period of continuous balance as the longest period of whole-body sway position remaining inside the virtual position limits. This time was computed over 60-s intervals for training trials and over the 30 s pre-learning and post-learning trials. Across all experiments, baseline (no delay) trials were analyzed in the same manner as delay trials.

### Postural oscillations, learning magnitudes, and learning rates

To estimate each participant’s level of balance performance for statistical comparisons, we extracted average sway velocity variance, average ankle torque variance and average longest time within limits from the first and last 5 min of training for each participant. The values from the last 5 min provided a comparison of the overall postural stability achieved by each training group at the end of training. To estimate the relative amount of learning (i.e., normalized learning magnitude) in each group, we computed the percent change (i.e., delta) between the first and last 5 min of training for each participant. This normalizing step was only used for sway velocity variance and ankle torque variance because the longest time within limits is already normalized by its maximum value. To quantify each participant’s learning rate, we fit first order exponential functions to the sway velocity variance, ankle torque variance and the longest time within the balance limits data obtained over the 40 min of training. Learning rates were defined as the time constants (time at 63.2% change) from the exponential fits.

## Statistical analysis

All statistical tests were performed using SPSS22 software (version 23.0, IBM) and the significance level was set to 0.05. Group data in the text and figures are presented as mean ± SEM. unless otherwise specified. Effect sizes (presented alongside ANOVA results) were calculated using the partial eta squared method.

### Training performance and learning

First, to evaluate the effects of older age and vision on baseline standing behavior before delay training, we compared the average sway velocity variance, ankle torque variance and longest time within limits from young and older participants with their eyes open and eyes closed. We performed separate factorial ANOVAs on these variables during baseline standing trials, with vision as a within-subject factor and age as a between-subject factor. To analyze the training data, we first determined whether each group managed to improve their balance performance during training by comparing responses from the first and last 5-min of training for average sway velocity variance, average ankle torque variance and average longest time within limits using one-tailed paired t-tests (Bonferonni corrected for multiple comparisons). We then ran several ANOVAs to test our hypotheses that (1) older adults would demonstrate reduced learning compared to young adults, and (2) learning differences would be influenced by the presence (or absence) of vision. For this analysis, we first tested whether there were differences across groups in overall postural stability achieved by the end of training with delays by running two-way between-subjects ANOVAs (between-subject factors: age, vision) on the sway velocity variance, ankle torque variance and longest time within limits from the last 5 min of training as dependent variables. Next, we performed two-way between-subject ANOVAs (between-subject factors: age, vision) using normalized learning magnitudes (i.e., deltas) and learning rates (i.e., time constants). This resulted in five separate ANOVAs performed using the following dependent measures: normalized learning magnitudes for sway velocity variance, normalized learning magnitudes for ankle torque variance, time constants from sway velocity variance fits, time constants from ankle torque variance fits, and time constants from longest time within limits fits. Note, time constants were identical for normalized and non-normalized fits.

### Transfer of learning between vision and no vision balance

We further investigated whether training to balance with or without vision transfers to the untrained visual condition. From the pre-learning and post-learning delay trials, we extracted balance behavior from sway velocity variance, ankle torque variance, and longest time within the balance limits and compared across trained and untrained conditions. For sway velocity variance and ankle torque variance, we calculated the percentage improvement between pre-and post-learning trials for both trained and untrained visual conditions. Pre-and post-learning sway velocity variance and ankle torque variance values were estimated from the two 30 s trials from each condition. For longest time within the limits, we compared how the longest time participants balanced within the limits in the post-learning trials differed between trained and untrained visual conditions. Because pre-and post-delay balance trials were only 30 s in length (repeated twice per condition), the maximum time within the limits was 30 s and was taken from the trial of best performance. To determine if participants transferred their improvements from the trained visual condition to the untrained visual condition, we compared the percent improvements in sway velocity variance and ankle torque variance (i.e., from pre- to post-) in the trained and untrained conditions using two-tailed paired t-tests (Bonferroni corrected). For longest time within limits we compared the values from trained and untrained conditions using two-tailed paired t-tests (Bonferroni corrected).

## Results

### Balance behavior in baseline standing

Participants first performed 60-s standing trials in baseline conditions (~4 ms delay), all participants were able to maintain a steady standing posture with only small oscillations in whole-body angle and without exceeding the position limits (see representative data in Figure 1C). Young adults had an average sway velocity variance of 0.04 ± 0.01 (°/s)^2^ and an average ankle torque variance of 4.55 ± 1.99 (Nm)^2^ vision trials which increased to 0.13 ± 0.03 (°/s)^2^ and 11.55 ± 3.22 (Nm)^2^ in no vision trials, respectively. Older adults exhibited an average sway velocity variance of 0.09 ± 0.04 (°/s)^2^ and an average ankle torque variance of 10.18 ± 1.72 (Nm)^2^ for vision baseline vision trials which increased to 0.20 ± 0.03 (°/s)^2^ and to 26.44 ± 9.13 (Nm)^2^ for no vision trials, respectively. A factorial ANOVA (within-subjects factor: vision; between-subjects factor: age) on sway velocity variance during baseline trials demonstrated that there were main effects of age [*F*_(1,38)_ = 5.23, *p* = 0.028, η_p_^2^ = 0.121] and vision [*F*_(1,38)_ = 8.32, *p* = 0.006, η_p_^2^ = 0.180] such that postural oscillations in baseline trials were larger in older adults and increased from vision to no vision trials. An identical factorial ANOVA on ankle torque variance during baseline trials demonstrated no main effect of age [*F*_(1,38)_ = 3.37, *p* = 0.074] but a main effect of vision [*F*_(1,38)_ = 7.06, *p* = 0.011, η_p_^2^ = 0.157]. These results align with widely-observed findings in the literature demonstrating that during quiet standing (1) older adults have greater postural sway variability than their young counterparts (Lord et al., 2018; Roman-Liu, 2018) and (2) removing vision for both age groups increases postural oscillations (Lord et al., 1991; Day et al., 1993; van der Kooij et al., 2011; Roman-Liu, 2018). Notably, there was no significant interaction between age and visual conditions for either sway velocity variance or ankle torque variance (both *p* > 0.05).

### Older age reduces the magnitude and rate of learning while removing vision has limited influence on postural oscillations

**Figure 2:**
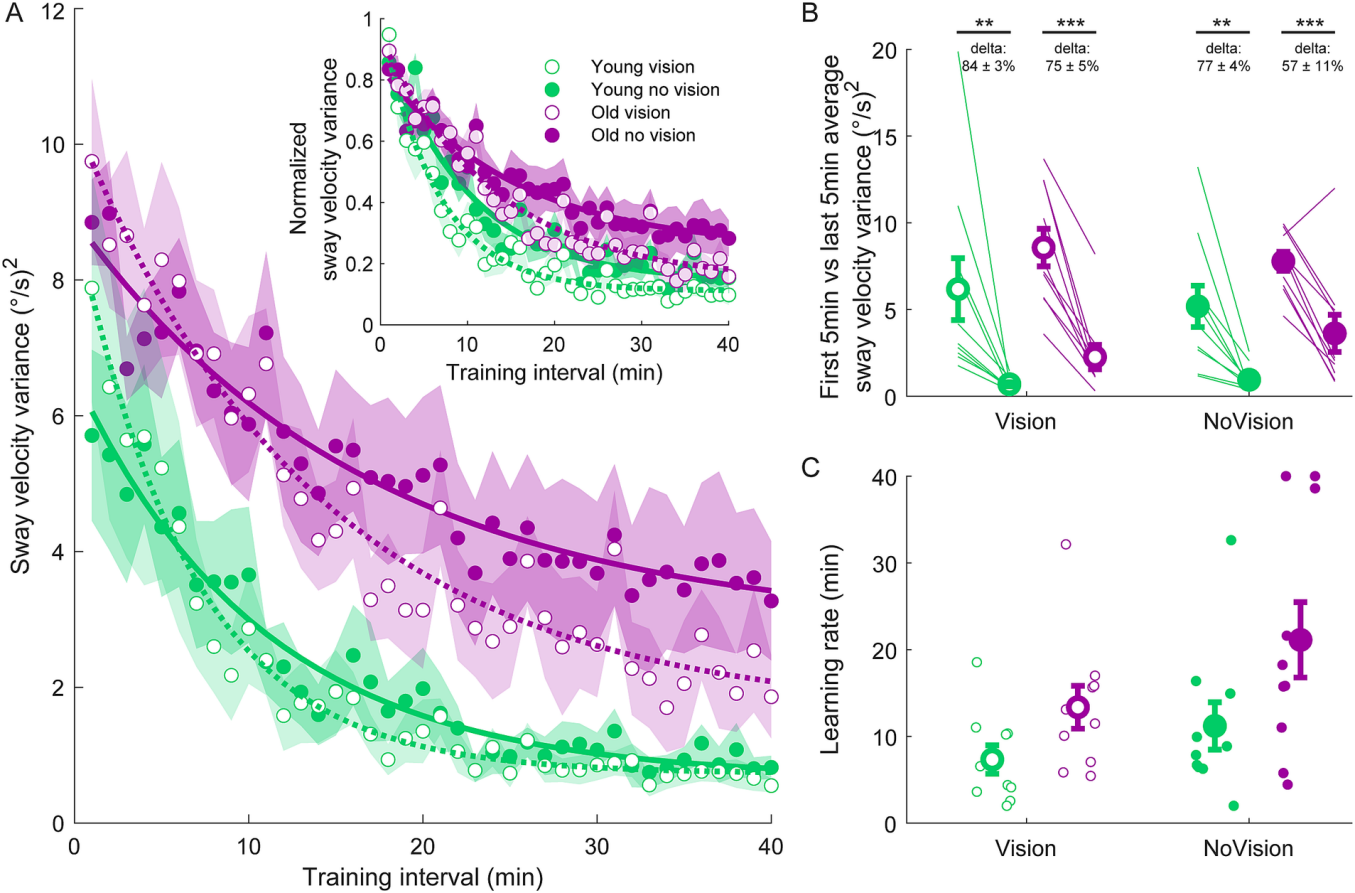
Sway velocity variance training data from all groups. **(A)** Average sway velocity variance estimated over 1-min intervals during the delay training protocol (*n* = 10 per group). Inset represents the normalized sway velocity variance. Exponential curves were fit to sway velocity variance data on a participant-by-participant basis. The curves presented are fitted to average sway velocity variance data for illustrative purposes. Curves were fit to a first order exponential function using a least-square method: $f\left(x\right)=a*\exp\left(\frac{-x}{b}\right)+c\text{.}$ Shaded regions represent SEMs for the average data points. **(B)** Average sway velocity variance extracted from the first and last 5 min of training (left and right markers, respectively) for all groups. Thin lines are individual participants and large circles are group averages (*n* = 10 for each group) with SEMs. Regardless of the training group, significant reductions in sway velocity variance were observed. Statistical tests depicted here represent paired t-tests between first and last 5 min of training. Delta values represent average normalized learning magnitude (i.e., delta or percentage change) from the first to last 5 min of training with SEMs. **(C)** Time constants extracted from exponential curves fitted to the sway velocity variance of each participant. Note, time constants from the non-normalized and normalized sway velocity variance fits were identical. Small circles represent individual participants and larger circles are group averages with SEMs. ** indicates *p* < 0.01 and *** indicates *p* < 0.001. Legend depicts color and symbol coding for groups.

**Figure 3:**
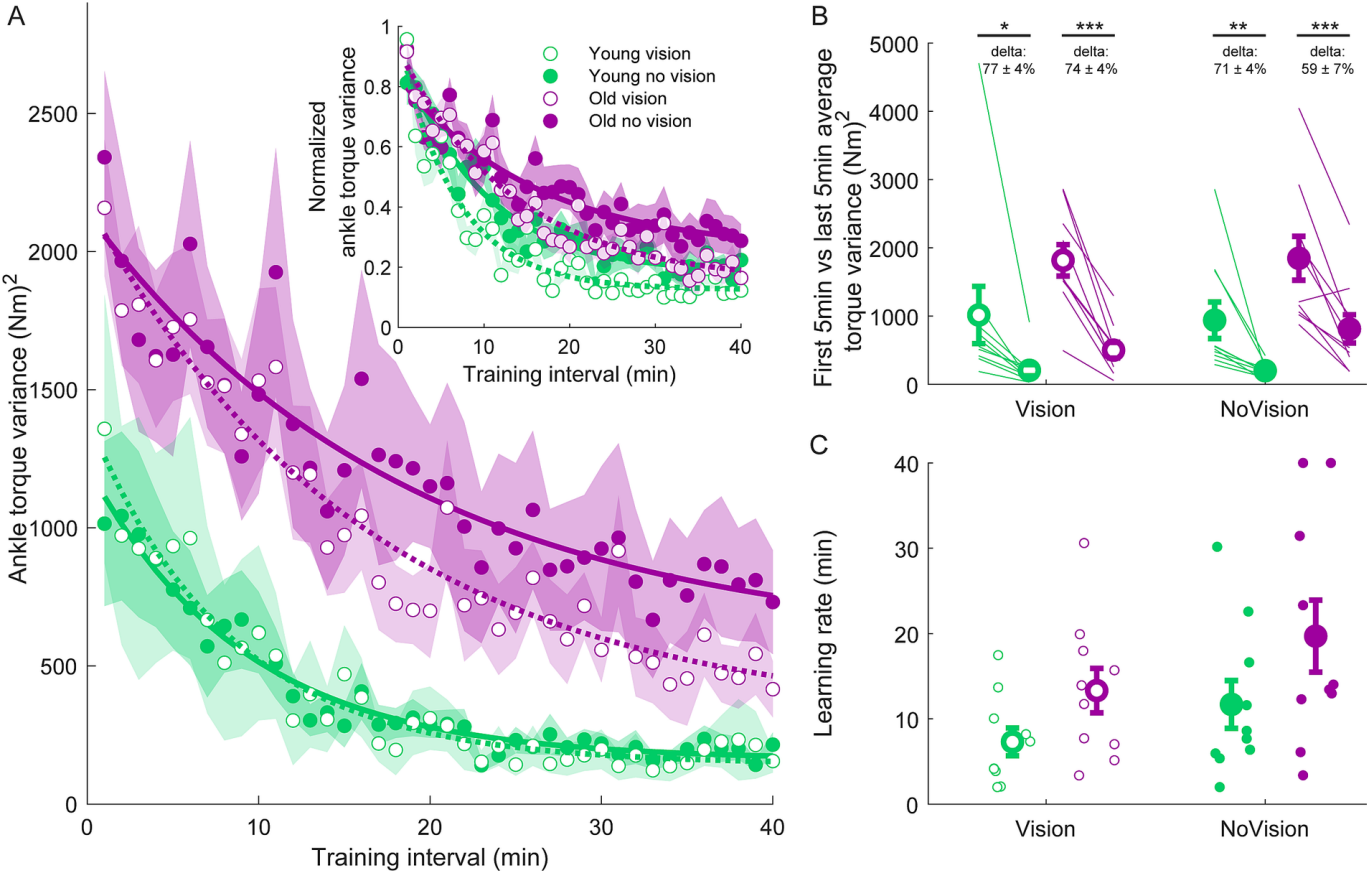
Ankle torque variance training data from all groups. **(A)** Average ankle torque variance estimated over 1-min intervals during the delay training protocol (*n* = 10 per group). Inset represents the normalized ankle torque variance. Exponential curves were fit to ankle torque variance data on a participant-by-participant basis. The curves presented are fitted to average ankle torque variance data for illustrative purposes. Curves were fit to a first order exponential function using a least-square method: $f\left(x\right)=a*\exp\left(\frac{-x}{b}\right)+c\text{.}$ Shaded regions represent SEMs for average data points. **(B)** Average ankle torque variance extracted from the first and last 5 min of training (left and right markers, respectively) for all groups. Thin lines are individual participants and large circles are group averages (*n* = 10 for each group) with SEMs. Regardless of the training group, significant reductions in ankle torque variance were observed. Statistical tests depicted here represent paired t-tests between first and last 5 min of training. Delta values represent average normalized learning magnitude (i.e., delta or percentage change) from the first to last 5 min of training with SEMs. **(C)** Time constants extracted from exponential curves fitted to the ankle torque variance of each participant. Note, time constants from the non-normalized and normalized torque variance fits were identical. Small circles represent individual participants and larger circles are group averages with SEMs. *indicates *p* < 0.05, ** indicates *p* < 0.01, and *** indicates *p* < 0.001. Legend depicts color and symbol coding for groups.

**Figure 4:**
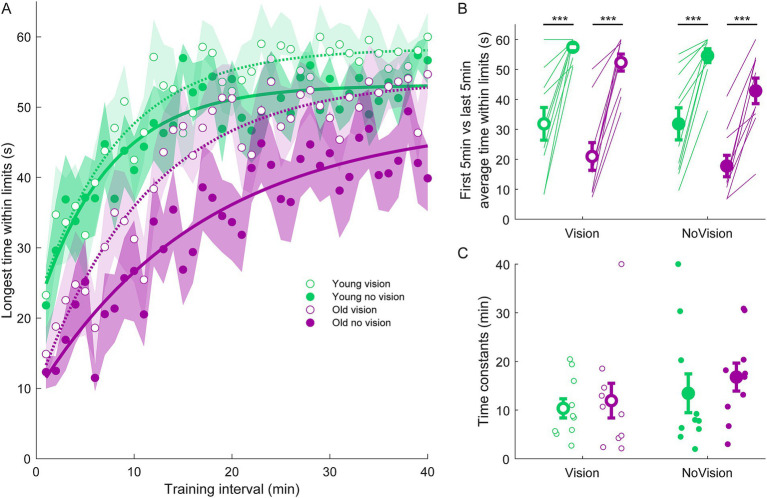
Longest continuous time within balance limits from all groups. **(A)** Average continuous time within limits estimated over 1-min intervals during the delay training protocol (*n* = 10 per group). Curves were fit to a first order exponential function using a least-square method: $f\left(x\right)=a*\exp\left(\frac{-x}{b}\right)+c\text{.}$ The curves presented are fitted to average longest time within limits for illustrative purposes. Shaded region represents SEMs for average data points. **(B)** Average longest time within limits from extracted from the first and last 5 min of training for all groups. Thin lines are individual participants and large circles are group averages (*n* = 10 for each group) with SEMs. Regardless of the training group, significant increases in the continuous time balancing within the limits were observed. Statistical tests depicted here represent paired t-tests between first and last 5 min of training. **(C)** Time constants extracted from longest time within limits curves. Small circles represent individual participants and larger circles are group averages with SEMs. *** indicates *p* < 0.001. Legend depicts color and symbol coding for groups.

Participants then performed four 30-s standing trials (with or without vision, each condition performed twice) with a 250 ms delay imposed between ankle-produced torques and accompanying whole-body motion. Pre-learning data from a representative participant in the y*oung vision group* is shown in Figure 1C. In these initial delay trials, participants swayed with high variability and had difficulty balancing within the position limits for more than a few seconds, as found previously (Rasman et al., 2021). Over the course of training, participants in all groups progressively improved their balance behavior, reducing their sway velocity variance (see Figure 2A) and ankle torque variance (see Figure 3A) while balancing for longer periods within the limits (see Figure 4A).

To examine learning within each group, we extracted average sway velocity variance, average ankle torque variance and average longest time within limits from the first and last 5 min of training for each participant. When comparing these two time points, we saw dramatic improvements in balance behavior in all groups (see Figures 2B, 3B, 4B). The *young vision group* reduced sway velocity variance from 6.17 ± 1.78 (°/s)^2^ to 0.69 ± 0.11 (°/s)^2^ (*p* < 0.01), reduced torque variance from 1016.21 ± 418.75 (Nm)^2^ to 205.60 ± 82.30 (Nm)^2^ (*p* < 0.05) and increased time within limits from 31.9 ± 5.5 s to 57.5 ± 1.2 s (*p* < 0.001). The *young no vision group* reduced sway velocity variance from 5.18 ± 1.19 (°/s)^2^ to 0.95 ± 0.24 (°/s)^2^ (*p* < 0.01), reduced torque variance from 939.25 ± 268.01 (Nm)^2^ to 200.07 ± 33.46 (Nm)^2^ (*p* < 0.01) and increased time within limits from 31.9 ± 5.4 s to 54.6 ± 2.3 s (*p* < 0.001). Similarly, the *old vision group* reduced sway velocity variance from 8.57 ± 1.08 (°/s)^2^ to 2.26 ± 0.70 (°/s)^2^ (*p* < 0.001), reduced torque variance from 1817.42 ± 230.57 (Nm)^2^ to 500.83 ± 115.65 (Nm)^2^ (*p* < 0.001) and increased time within limits from 21.0 ± 4.6 s to 52.3 ± 2.8 s (*p* < 0.001). Finally, the *old no vision group* reduced sway velocity variance from 7.78 ± 0.55 (°/s)^2^ to 3.62 ± 1.07 (°/s)^2^ (*p* < 0.001), reduced torque variance from 1847.61 ± 321.38 (Nm)^2^ to 813.39 ± 207.53 (Nm)^2^ (*p* < 0.001) and increased time within limits from 17.8 ± 3.6 s to 42.9 ± 4.3 s (*p* < 0.001).

Comparison of these metrics across the different groups in the last 5 min of training indicated that the *young vision group* had the lowest postural oscillations while the *old no vision group* had the highest oscillations (see Table 1). A two-way between subjects ANOVA partially confirmed these observations, showing that for sway velocity variance in the last 5 min of training, there was a main effect of age, no effect of vision and no interaction between age and vision (see Table 1). An identical ANOVA on ankle torque variance from the last 5 min of training showed a main effect of age, no effect of vision and no interaction between age and vision (Table 1). Finally, a two-way between subjects ANOVA on the average longest time within balance limits from the last 5 min of training showed a main effect of age and a main effect of vision, but no interaction between variables (Table 1). These results reveal that young adults demonstrated reduced whole-body oscillations compared to older adults by the end of training, and that participants who train to balance with vision can better remain within the balance limits compared to those who train without vision.

**Table 1:** Summary of balance improvement metrics from training and their statistical tests.

Dependent variable	Young vision	Old vision	Young no vision	Old no vision	ANOVA main effects and interaction					
					Age		Vision		Age × Vision interaction	
					F_1,36_	*P*	F_1,36_	*P*	F_1,36_	*P*
Average from last 5 min of training										
Sway velocity variance (°/s)^2^	0.69 ± 0.11	2.26 ± 0.70	0.95 ± 0.24	3.62 ± 1.07	10.51	*0.003*	1.54	0.223	0.711	0.405
Ankle torque variance (Nm)^2^	205.60 ± 82.30	500.83 ± 115.65	200.07 ± 33.46	813.39 ± 207.53	12.83	*0.001*	1.47	0.234	1.57	0.218
Longest time within limits (s)	57.5 ± 1.2	52.3 ± 2.8	54.6 ± 2.3	42.9 ± 4.3	8.61	*0.006*	4.59	*0.039*	1.34	0.256
Learning magnitudes										
Sway velocity variance (% change)	84 ± 3	75 ± 5	77 ± 4	57 ± 11	5.12	*0.030*	3.80	0.059	0.773	0.385
Ankle torque variance (% change)	77 ± 4	74 ± 4	71 ± 4	59 ± 7	2.28	0.140	4.62	*0.038*	0.757	0.390
Learning rates										
Sway velocity variance (min)	7.3 ± 1.6	13.4 ± 2.5	11.2 ± 2.7	21.1 ± 4.3	7.24	*0.011*	3.85	0.058	0.433	0.515
Ankle torque variance (min)	7.3 ± 1.6	13.3 ± 2.6	11.7 ± 2.8	19.7 ± 4.2	5.61	*0.023*	3.31	0.077	0.113	0.738
Longest time within limits (min)	10.4 ± 1.9	11.9 ± 3.6	13.5 ± 4.0	16.8 ± 2.9	0.592	0.447	1.55	0.222	0.074	0.787

For all dependent variables listed, a two-way between subjects ANOVA [between-subjects factors: age (i.e., young vs old); vision (training with vision vs training without vision)] was run. The italicized values represent significant effects.

Since older adults balanced with higher sway variability before training, it is possible that the relative amount of learning was in fact similar across age groups. Therefore, we computed the normalized learning magnitude (i.e., delta or percentage change) in sway velocity variance and ankle torque variance from the first and last 5 min of training. These learning magnitudes are depicted in Figures 2B, 3B (see deltas). For sway velocity variance, the largest normalized learning magnitude occurred in the *young vision group* (84 ± 3% reduction) while the smallest occurred in the *old no vision group* (57 ± 11% reduction). A two-way between subjects ANOVA revealed a main effect of age, no effect of vision and no interaction between age and vision (see Table 1). Similarly, for ankle torque variance the largest normalized learning magnitude occurred in the *young vision group* (77 ± 4% reduction) while the smallest occurred in the *old no vision group* (59 ± 7% reduction). A two-way between subjects ANOVA revealed no effect of age, an effect of vision and no interaction between age and vision (Table 1). These results demonstrate that compared to their younger counterparts, older adults show less relative learning (smaller learning magnitudes) when training to balance with imposed 250 ms delays.

Finally, to further characterize balance learning across groups, we also compared learning rates (i.e., time constants extracted from exponential fits) for sway velocity variance, ankle torque variance and continuous time within limits (Figures 2C, 3C, 4C). A two-way between subjects ANOVA with sway velocity variance learning rates indicated a main effect of age, no effect of vision and no interaction between age and vision (see Table 1). An identical ANOVA using learning rates from ankle torque variance curves showed a main effect of age, no effect of vision and no interaction between age and vision (Table 1). Finally, a two-way between subjects ANOVA using learning rates from continuous time within limits showed no significant effects of age or vision and no significant interaction between age and vision variables. Overall, older age significantly slowed learning rates (with the exception of time within limits) whereas vision did not significantly influence learning rates.

### Transfer of learning between visual conditions

**Figure 5:**
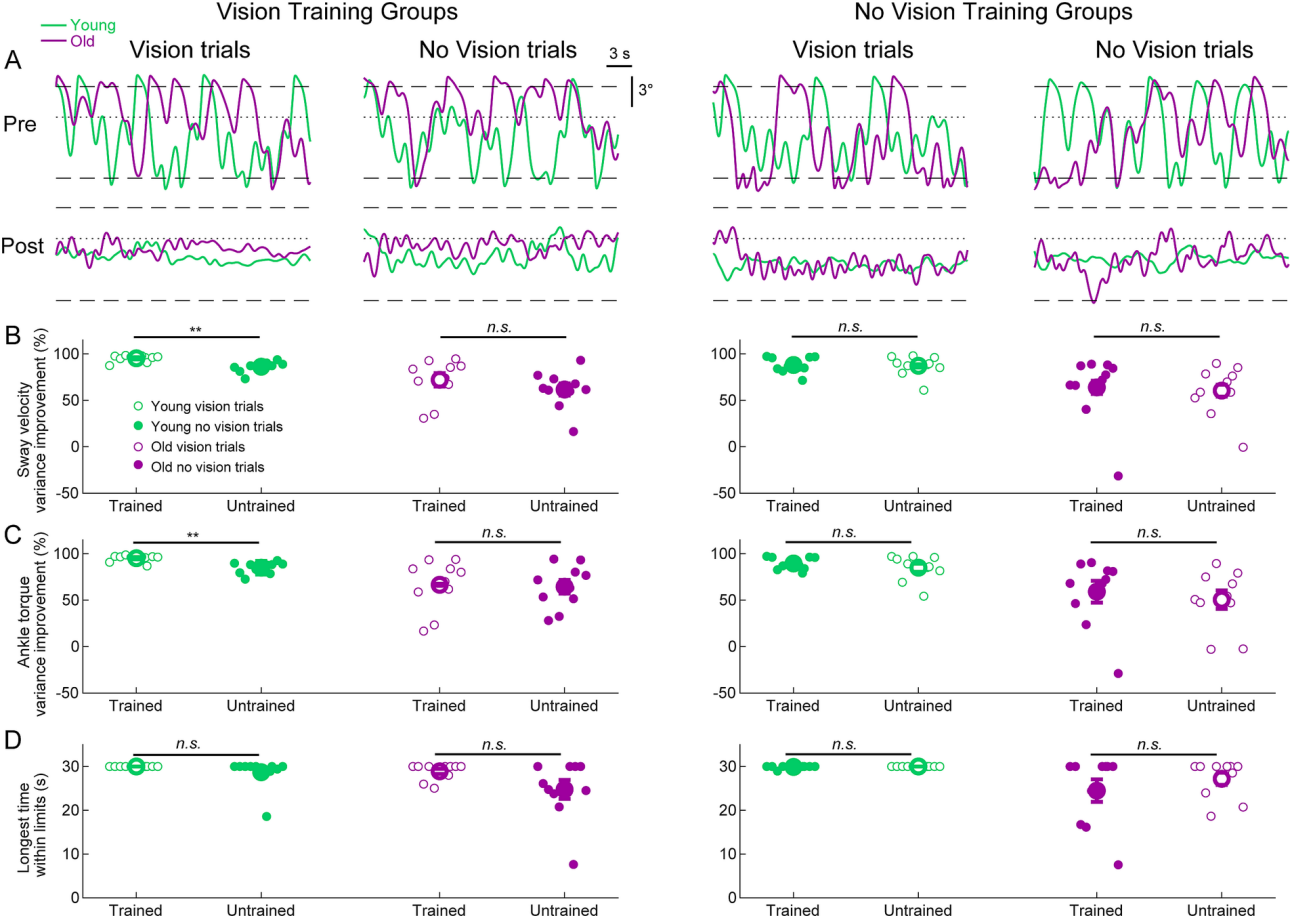
Standing balance behavior with imposed 250 ms delay in pre-and post-learning trials. **(A)** Whole-body angular position (°) from representative participants from all groups. Dashed lines represent the virtual position limits (6° anterior, 3° posterior) and the dotted lines represent 0°. For all panels, data in green represents young adults and data in purple represents old adults. **(B)** Sway velocity variance percentage improvements between pre-and post-learning trials from all groups. **(C)** Ankle torque variance percentage improvements between pre-and post-learning trials from all groups. **(D)** Longest time within balance limits in the pre-and post-learning trials. Pre-and post-learning delay trials were 30 s in length, resulting in a maximum value of 30 s for time within limits. For **(B–D)**, small circles are individual participants and large circles are group averages (*n* = 10 for each group) with SEMs. Open circles represent vision trials and closed circles represent no vision trials. Regardless of which visual condition was trained (vision or no vision), balance improvements were observed for both vision and no vision balance conditions. A significant difference between trained and untrained conditions indicates that the improvement percentages across trained and untrained visual conditions were equivalent. ** indicates *p* < 0.01, n.s. indicates not significant.

We further investigated whether training to balance with or without vision transfers to the untrained visual condition by examining the difference between pre-learning and post-learning delay balance trials (Figure 5A). For sway velocity variance and ankle torque variance, we present the percentage change (i.e., improvement) between pre-and post-learning trials for both trained and untrained visual conditions (Figures 5B,C). For longest time within the limits, we present the longest time they balanced within the limits in the post-learning trials for both trained and untrained conditions (Figure 5D and see Methods). To determine if participants transferred their improvements from the trained condition to the untrained conditions, we compared the trained and untrained improvement metrics (i.e., pre-post differences) using two-tailed paired t-tests. The only statistically significant differences between trained and untrained conditions occurred in the *young vision group* (see Figures 5B,C). Notably, sway velocity variance decreased (i.e., improved) by 95 ± 1% in the trained vision condition *vs* improving by 86 ± 2% in the untrained no vision condition (t_(9)_ = 4.60, *p* < 0.01). Similarly, ankle torque variance decreased (i.e., improved) by 95 ± 1% in the trained vision condition vs. improving by 85 ± 7% in the no vision untrained condition (t_(9)_ = 4.07, *p* < 0.01). Therefore, for these two metrics, participants in the *young vision group* transferred most but not all of their balance improvement to the untrained no vision condition. For all other comparisons in all groups, there were no differences in balance improvement between the trained and untrained conditions. Therefore, despite only training in one visual state, both young and older adults transferred nearly all of their balance improvements to the untrained visual state.

## Discussion

The primary aims of this study were to determine whether and how learning to balance with imposed sensorimotor delays is affected by (1) older age and (2) the absence of visual feedback. We used a robotic balance simulator to artificially increase sensorimotor delays in the human control of standing balance and trained young (< 30 years) and older participants (> 65 years) to balance with these novel delays. Participants practiced balancing with an imposed 250 ms delay either with or without vision. All participants, regardless of age or visual training condition, improved their balance performance through training to stand with the imposed delay. Compared to young adults, however, older adults had larger postural oscillations, exhibited less relative learning and had slower rates of balance improvement.

The reduced overall postural stability observed in older adults aligns with numerous other findings of older adults demonstrating increased variability in postural sway (Woollacott et al., 1986; Lin and Woollacott, 2002; Lord et al., 2018; Roman-Liu, 2018). Less understood in the literature, however, is whether older age impairs the ability to adapt and recalibrate balance when exposed to novel sensorimotor relationships in postural control. Our robotic simulator allowed us to address this question by imposing a controlled sensorimotor manipulation (Rasman et al., 2018), i.e., an imposed delay in balance control. Confirming our first hypothesis, we found that compared to their young counterparts, older adults had reduced balance learning to this novel sensorimotor environment. This impairment of balance learning expands on the previously reported reductions in sensorimotor learning in other motor tasks (e.g., upper limb reaching) (Seidler, 2006, 2007; Vandevoorde and Orban de Xivry, 2019; Wolpe et al., 2020). Contrary to our second hypothesis, removing vision did not significantly reduce relative learning magnitudes or slow learning rates, though it did partially limit the amount of time participants could remain within the balance limits. Furthermore, there was no interaction between age and the presence of visual feedback on these dependent measures. These results therefore do not support the expectation that removing visual feedback would impair balance learning for all participants and to a larger degree in older adults. Finally, we found that the learning acquired in both visual conditions transferred to the untrained condition for young and older participants, demonstrating generalization across different states of sensory feedback. Taken together, our study reveals that advanced age impairs balance learning, but importantly, older adults retain the ability to recalibrate balance control and learn to stand with long sensorimotor delays. Further, visual feedback is not required for young or older adults to relearn to stand with novel sensorimotor delays, and only has a limited effect on postural stability.

### Older adults can learn to stand with imposed sensorimotor delays but their balance is impaired compared to young adults

In the present study, we found both young and older adults could learn to maintain upright standing balance with imposed 250 ms delays, with or without vision. Older adults, however, had larger postural oscillations, reduced learning magnitudes and learned at slower rates. For young adults, sensorimotor delays associated with upright balance control are estimated to be ~100–160 ms (Kuo, 1995; van der Kooij et al., 1999; Bingham et al., 2011). When these delays are artificially increased in young adults, whole-body oscillations increase such that maintaining posture within the balance limits is difficult to achieve beyond imposed delays of ~200 ms (i.e., net delays of ~300–360 ms) and becomes increasingly difficult as the delay increases (Rasman et al., 2021). For older adults, natural aging increases physiological sensorimotor delays due to changes in nerve conduction, muscle force generation and neural processing (Dorfman and Bosley, 1979; Eyre et al., 1991). As a result, balance responses to postural perturbations occur 10–30 ms later in adults ≥60 years old (Lin and Woollacott, 2002; Davidson et al., 2011; Wiesmeier et al., 2015). In our experiments, the increased oscillations, and perhaps the reduced learning, observed in older adults may have been partially due to net sensorimotor delays (physiological plus robot-induced) being longer for older adults compared to their young counterparts.

In addition to having to overcome longer sensorimotor delays, there are a variety physiological and cognitive factors that may also impair balance learning in older adults. First, in non-balancing motor tasks, sensorimotor learning has been reported to be diminished in older adults (Seidler, 2007; Wolpe et al., 2016; Vandevoorde and Orban de Xivry, 2019; Vachon et al., 2020) and linked to changes in brain structure and central processing (Seidler et al., 2010; Wolpe et al., 2020). These physiological and morphological changes may further influence sensorimotor learning in balance control. Second, the acuity of balance-relevant sensory cues (vestibular, visual, somatosensory, auditory) is known to degrade with older age (Shaffer and Harrison, 2007; Patel et al., 2009; Lord et al., 2018; Wagner et al., 2023). This decrease in sensory acuity may limit the nervous system’s ability to re-associate unexpected sensory feedback with balancing motor actions (Seidler et al., 2010; Wolpe et al., 2016). Third, older age is associated with reduced muscular strength (Larsson et al., 1979; Kallman et al., 1990; Doherty, 2003), slower rates of force development (Larsson et al., 1979; Thelen et al., 1996) and volitional reaction times (Lord and Fitzpatrick, 2001). These factors may compromise the ability to generate the motor commands needed to balance with long robot-induced delays. Finally, the decrease in cognitive contributions to motor control that accompanies advanced age can negatively affect explicit (i.e., cognitive) learning mechanisms (Vandevoorde and Orban de Xivry, 2019; Vachon et al., 2020; Wolpe et al., 2020). While it is not clear how explicit mechanisms (conscious awareness and cognitive strategies) and implicit mechanisms (i.e., automatic and absent of cognition) contribute to learning to balance with control delays, it is expected that any reliance on explicit mechanisms will have a more substantial impact on older adults.

### Visual feedback is not critical for learning to balance with novel sensorimotor delays

In our experiments, we found that both young and older participants could learn to maintain upright balance for prolonged periods (< 60 s) while standing with long sensorimotor delays with or without visual feedback. Although training with vision slightly improved balance performance (i.e., longer periods of balancing within the limits at the end of training), participants who trained without vision could still adapt and learn to balance in the novel task. In our previous work on delayed balance learning, visual, vestibular and somatosensory signals of whole-body position and motion were available while young participants balanced and trained with imposed delays (Rasman et al., 2021). Therefore, our current findings imply that both the young and older nervous system can rely on self-motion estimates derived from only vestibular and somatosensory cues to recalibrate balance control.

Why does vision only appear to play a moderate role in learning to stand with unexpected sensorimotor delays? This may be due to several reasons regarding the context of the balance task. First, compared to the sensorimotor loops comprising vestibular and somatosensory signals, the visuomotor system is associated with greater physiological delays in nerve conduction and central processing (Franklin et al., 2008; Franklin and Wolpert, 2011). Consequently, visual stimuli delivered while standing evoke balance responses with latencies >100 ms longer than those evoked by vestibular or somatosensory inputs (Day et al., 1997; Guerraz et al., 2001; Day and Guerraz, 2007). This slower processing of visual signals may result in visual feedback being less relevant compared to other sensory cues for correcting whole-body movements while balancing with long robot-imposed sensorimotor delays. Second, because visual input provides crucial information about the surrounding world (i.e., exteroceptive cues), it may be more relied upon for learning in balance tasks that are dependent on explicitly orienting the body with respect to the environment, or responding to externally imposed visual disturbances (Paulus et al., 1984; Dokka et al., 2010; Day et al., 2016). Indeed, many previous studies that suggest older adults have greater dependence on visual inputs for balance arrived at this conclusion by observing larger evoked responses to visual perturbations (Sundermier et al., 1996; Bugnariu and Fung, 2007; Jeka et al., 2010). Importantly, while externally imposed perturbations can reveal changes in control necessary to address unexpected disturbances, the observed adaptations differ from the sensorimotor recalibrations linked to learning novel control dynamics (Rasman et al., 2018). By altering the relationships between the motor commands and sensory feedback of balance (such as imposing delays), the brain is compelled to remap the sensory consequences of a desired balance behavior to improve control. As a result, we do not interpret our findings as contradicting earlier reports of older adults having larger balance responses to visual perturbations. Instead, we view our findings as revealing the adaptive capabilities of the young and older balance system under varying sensory conditions. Our work builds on studies showing that learning novel upper limb dynamics can occur without vision (DiZio and Lackner, 2000; Franklin et al., 2007; Marko et al., 2012) and reveal that learning to balance the whole body with delays is also possible without vision.

In addition to the balance learning that occurred in both visual conditions, we observed that regardless of which condition was trained, all participants transferred their training benefits to the untrained visual condition. This indicates that the brain can generalize learned balance control across different sensorimotor contexts and swiftly adjust balance while transitioning between states. These results expand on our previous findings that learned balance control with sensorimotor delays generalizes across (1) delay lengths (Rasman et al., 2021), (2) movement directions and (3) muscle effectors (Rasman et al., submitted). The ability to transfer learned control policies between tasks is influenced by the similarity and differences of contextual factors (i.e., task goal, environment, available sensory cues) across trained and untrained tasks (Berniker and Kording, 2008; Braun et al., 2010; de la Malla et al., 2014). In the present study, the primary factor that varied between conditions was the available sensory cues relied on to control balance (visual, vestibular, somatosensory *vs* vestibular and somatosensory only). Given the strong transfer of learning observed, it appears the brain can recognize the contextual similarities between these balance tasks and readily generalize learned control. Generalizing learned balance skills to different sensorimotor contexts is important for individuals to adapt and maintain stable balance control across familiar and novel environments. In turn, balance training and rehabilitation interventions need to evoke generalizable learning to be useful for daily postural activities (Roemmich and Bastian, 2018; Harper et al., 2021). Combined with our previous findings (Rasman et al., 2021; Rasman et al., submitted), our present results encourage the exploration of future robot-assisted therapies that evoke generalizable improvements in balance function.

### Implications for training and rehabilitation

Although age-related physiological and cognitive changes may limit learning, most older adults in our study learned to maintain upright posture (i.e., not falling for at least 60 s) while balancing with the delay. These adaptive capabilities are encouraging for training and rehabilitative therapies that aim to improve balance control and reduce the risk of falling in older populations. We have previously demonstrated that after training with imposed delays, young adults show long-term retention (tested ~3 months later) of balance learning and that learning generalizes across sensorimotor delays of different magnitudes (Rasman et al., 2021). Combining these previous results with our present findings, it is possible to envision training and rehabilitative therapies using similar robotic interventions to improve balance function in older adults and clinical populations with balance deficits (e.g., multiple sclerosis, cerebellar ataxia). Importantly, the robotic system provides an environment where individuals can train in challenging balance conditions without the risk of falling (i.e., the robot physically prevents one from falling if balance is lost). This allows individuals to safely explore and learn different balance control strategies that may be useful in daily bipedal activities. Our study was limited, however, because we did not test whether the improvement in balance function through training with induced delays extends to everyday postural activities. Future work should explore this possibility.

### Limitations and other considerations

We note that our study was not designed to investigate the relative contributions of implicit (automatic, absent of cognition) and explicit (conscious awareness) mechanisms to learn to balance with long sensorimotor delays. Over the last decade, this topic has received much interest in the field of motor control (McDougle et al., 2016; Krakauer et al., 2019) and recent studies have focused on determining how the contribution of these mechanisms change with aging (Vandevoorde and Orban de Xivry, 2019; Vachon et al., 2020; Wolpe et al., 2020). Implementing experiments that compare and contrast balance learning to short-imperceptible delays and long-perceptible delays may facilitate the exploration of implicit and explicit learning mechanisms, respectively. Furthermore, providing participants with explicit instruction on how to improve their balance (which was not provided in the present study) may provide insight into how young and older adults use explicit knowledge to learn novel balance tasks.

## Conclusion

Our study demonstrates that while older age impairs human learning to balance with unexpected sensorimotor delays, both young and older adults can learn to maintain upright stance with imposed delays of 250 ms (net ~350–440 ms). We further demonstrate that visual feedback is not crucial for learning to stand with long sensorimotor delays. Finally, both young and older adults can learn to balance with or without vision and transfer learned balance control to the untrained visual state. Overall, while advanced age partially impairs balance learning, the older nervous system maintains the ability to recalibrate motor control to stand with initially destabilizing sensorimotor delays under differing visual conditions.

## Data availability statement

The raw data supporting the conclusions of this article will be made available by the authors, upon reasonable request.

## Ethics statement

The studies involving humans were approved by the Medical Research Ethics Committee Erasmus MC. The studies were conducted in accordance with the local legislation and institutional requirements. The participants provided their written informed consent to participate in this study. Written informed consent was obtained from the individual(s) for the publication of any potentially identifiable images or data included in this article.

## Author contributions

BGR: Conceptualization, Formal analysis, Investigation, Methodology, Project administration, Software, Validation, Visualization, Writing – original draft, Writing – review & editing. CZ: Conceptualization, Formal analysis, Investigation, Methodology, Software, Visualization, Writing – review & editing. PAF: Conceptualization, Data curation, Funding acquisition, Investigation, Methodology, Project administration, Resources, Software, Supervision, Validation, Visualization, Writing – review & editing.
